# Exploring the Link Between COVID-19 and Liver Abscess Formation: A Tertiary Care Center-Based Analysis of Pathophysiological Insights and Clinical Implications

**DOI:** 10.7759/cureus.94136

**Published:** 2025-10-08

**Authors:** Ramlakhan S Verma, Shailendra Pal Singh, Gaurav Mishra, Vikas K Patel, Vipin Gupta

**Affiliations:** 1 General Surgery, Uttar Pradesh University of Medical Sciences, Etawah, IND; 2 General Surgery, Uttar Pradesh University Of Medical Sciences, Etawah, IND

**Keywords:** comorbidities, covid-19, inflammation, liver abscess, risk factors

## Abstract

Background

Liver abscess is a potential concern among liver health complications. With severe acute respiratory syndrome coronavirus 2 (SARS‑CoV‑2) capable of inducing inflammation and immune dysfunction, this study aimed to examine the association between COVID-19 infection and liver abscess risk.

Methods

This prospective study was conducted at Uttar Pradesh University of Medical Sciences (UPUMS), Saifai, Etawah, India. A sample of 150 patients with radiologically diagnosed liver abscesses was enrolled. Patient data, including COVID-19 history, clinical characteristics, and comorbidities, were collected, and laboratory parameters were analyzed, including inflammatory markers like C-reactive protein (CRP) and interleukin 6 (IL-6).

Results

The average age of participants was 48.3 years, with a male preponderance (73.3%). Among those with liver abscesses, 60% had a history of COVID-19, and of these, 43.3% required hospitalization, with 20% needing ICU care. High prevalence rates of diabetes (40%) and liver cirrhosis (23.3%) were noted. Percutaneous drainage was the most common treatment, achieving a recovery rate of 80%. Elevated inflammatory markers were significantly associated with COVID-19 severity and liver abscess risk.

Conclusion

The study highlighted that severe COVID-19, especially in patients with diabetes or liver cirrhosis, increases the risk of liver abscess. Routine monitoring for liver health in these patients is recommended.

## Introduction

The COVID-19 pandemic has highlighted a wide range of health challenges that extend beyond just respiratory symptoms, drawing attention to potential complications affecting liver health. Among these, liver abscesses, particularly pyogenic and amoebic types, have been reported in some COVID-19 cases, suggesting a possible link that requires thorough investigation [1,2]. Severe acute respiratory syndrome coronavirus 2 (SARS‑CoV‑2), the virus responsible for COVID-19, has demonstrated the ability to directly affect liver tissues, often causing abnormalities in liver enzyme levels, inflammation, and, in some instances, the presence of the virus within hepatic cells [3-5]. Although studies have documented liver injury in COVID-19 patients, the exact mechanisms that may contribute to liver abscess formation remain largely speculative [6].

Typically, liver abscesses develop due to bacterial or parasitic infections spreading to the liver, either through the bloodstream or by direct extension from adjacent areas. In COVID-19 cases, however, compromised immune function, excessive inflammation, and direct liver damage may further increase this risk. Case studies and autopsies have identified immune response activity and inflammatory reactions in the liver tissues of COVID-19 patients, suggesting pathways that could facilitate abscess formation [7,8]. Moreover, severe immune responses, including cytokine storms observed in COVID-19, could heighten vulnerability to secondary infections, potentially involving the liver [9]. Nevertheless, the progression from viral liver injury to abscess formation has not been fully explored or understood [10,11]. COVID-19 has also been associated with multiple severe complications, including vascular events, such as pulmonary embolism [12], which may exacerbate systemic inflammation and contribute indirectly to hepatic injury.

## Materials and methods

This prospective study was conducted in the Department of General Surgery at Uttar Pradesh University of Medical Sciences (UPUMS), starting in September 2022, with the goal of investigating the association between COVID-19 infection and liver abscess formation, along with an analysis of emerging risk factors. A sample of n=150 patients diagnosed with liver abscess through clinical and radiological evaluation was recruited based on specific inclusion and exclusion criteria.

Inclusion criteria included patients with a confirmed liver abscess, while exclusion criteria omitted those with co-existing liver tumors, hepatic cysts, hydatid cysts, or liver metastasis to ensure focus on liver abscess cases potentially linked to COVID-19 infection.

Patient data collection

Detailed data on each patient’s demographics, history of COVID-19, medical background, and lifestyle factors were collected. Specific COVID-19-related information included the severity of prior infection (categorized as mild, moderate, or severe), duration since infection, and any treatments received, such as antiviral medications, steroids, or immunosuppressants.

These factors were documented to examine any associations between COVID-19 severity or treatment and the development of liver abscesses.

Data on additional lifestyle factors, such as alcohol and tobacco use, socioeconomic status, and occupation, were recorded to explore broader risk factors. Nutritional status was assessed via body mass index (BMI), serum albumin, and hemoglobin levels, as nutritional health may influence liver abscess susceptibility.

Clinical assessment and diagnostic tools

A comprehensive clinical assessment included documenting patient age, sex, history of diabetes mellitus, liver cirrhosis, immunosuppression, and previous liver conditions. Radiological investigations, including ultrasound (USG) and contrast-enhanced computed tomography (CECT) of the abdomen, were used to confirm the diagnosis of liver abscess and to assess its size, number, and location. Liver elastography (FibroScan) was performed where feasible for an assessment of liver stiffness and to detect underlying fibrosis or liver damage.

Laboratory investigations

Standard laboratory tests were conducted to evaluate infection and liver health, including liver function test, CBC, and culture of blood. Inflammatory markers (e.g., C-reactive protein (CRP), interleukin-6 (IL-6), and D-dimer) and immune function tests were also included to assess immune dysregulation potentially linked to both COVID-19 and liver abscess formation.

Treatment and follow-up

Patients received standard management for liver abscess as per hospital protocols, including percutaneous drainage or surgical intervention when necessary. A follow-up period of six months post-diagnosis was established to monitor outcomes, recurrence, or any delayed liver complications, providing insights into COVID-19’s long-term effects on liver health.

Data management

Data was meticulously recorded in a secure electronic database, ensuring accuracy and completeness through regular validation checks and quality controls. Information on COVID-19 hospitalizations, ICU admissions, and ventilation status was documented, allowing for a deeper analysis of severe COVID-19 complications as potential risk factors.

Statistical analysis

Descriptive statistics have summarized patient demographics and clinical characteristics, while bivariate and multivariate analyses, including logistic regression models, assessed the association between COVID-19 and liver abscess development. Adjustments for confounding variables, such as age, pre-existing conditions, and lifestyle factors, were made to isolate COVID-19 as an independent risk factor. Sensitivity and subgroup analyses were conducted to address confounding effects and to validate the robustness of findings. Statistical significance was set as a priority, and analyses were conducted using statistical software to draw reliable conclusions on the role of COVID-19 and emerging risk factors in liver abscess formation.

## Results

Demographic, clinical, and COVID-19-related characteristics for the 150 patients are shown in Table 1. The average age was 48.3 ± 15.2 years, with a majority being male (110; 73.3%). Approximately 70 (46.7%) patients reported the use of alcohol, while 45 (30%) consumed tobacco. A significant portion of the participants (90; 60%) had a prior COVID-19 infection, with 65 (43.3%) requiring hospitalization and 30 (20%) needing ICU care. Key clinical factors included a 40% prevalence of diabetes and a 23.3% prevalence of liver cirrhosis, highlighting important risk considerations for liver abscesses in the post-COVID-19 context.

**Table 1:** Clinical and demographical variables of patients (n=150) with liver abscess

Variable	n or mean ± SD	%
Age (years)	48.3 ± 15.2	-
Gender		
- Male	110	73.3
- Female	40	26.7
Tobacco Use	45	30
Alcohol Use	70	46.7
History of COVID-19		
- Yes	90	60
- No	60	40
Severity of COVID-19 Infection		
- Mild	30	20
- Moderate	40	26.7
- Severe	20	13.3
Duration Since COVID-19 Recovery (months)	8.2 ± 3.5	-
COVID-19 Treatment		
- Antivirals	55	36.7
- Steroids	45	30
- Immunosuppressive Therapy	20	13.3
Hospitalization for COVID-19	65	43.3
ICU Admission	30	20
Ventilation Support	15	10
Diabetes Mellitus	60	40
Liver Cirrhosis	35	23.3
Immunosuppression	20	13.3
- HIV	10	6.7
- Immunosuppressive Drugs	10	6.7
History of Biliary Tract Disease	25	16.7
BMI (kg/m²)	26.4 ± 4.3	-
Nutritional Status		
- Malnutrition	40	26.7
- Normal Nutrition	110	73.3

Radiological and laboratory findings are shown in Table 2. Most cases had a single abscess on ultrasound 110 (73.3%), predominantly located in the right lobe. Elevated inflammatory markers, including CRP and IL-6, were also observed, reflecting infection-related responses.

**Table 2:** Key radiological and laboratory parameters of patients (n=150) with a liver abscess

	n (150)	%
Ultrasound (USG) Findings		
Number of Abscesses		
Single	110	73.3
Multiple	40	26.7
Size of Abscess (cm)	4.2 ± 1.5	-
Location (Liver Lobe)		
Right	100	66.7
Left	30	20
Both	20	13.3
Contrast-Enhanced CT (CECT) Abscess Characteristics		
Simple	115	76.7
Complex	35	23.3
Liver Fibrosis (FibroScan)		
Mild	90	60
Moderate	40	26.7
Severe	20	13.3
Complete Blood Count (CBC)	Mean ± SD	-
WBC (×10⁹/L)	12.4 ± 3.1	-
Hemoglobin (g/dL)	11.3 ± 1.8	-
Liver Function Tests (LFTs)		
AST (U/L)	78.5 ± 20.3	-
ALT (U/L)	82.1 ± 22.7	-
Total Bilirubin (mg/dL)	1.6 ± 0.4	-
Inflammatory Markers		
Serum C-Reactive Protien (mg/L)	48.3 ± 15.4	-
Serum Interlukin-6 (IL-6) (pg/mL)	32.1 ± 8.9	-
D-dimer (ng/mL)	520 ± 150	-

Of the 150 patients assessed for prior COVID-19 infection history, associations with postoperative outcomes were analyzed using Spearman’s rank correlation coefficient. Prior COVID-19 infection was found to have a statistically significant, though weak, positive correlation with postoperative mortality (Spearman’s ρ = 0.086, df = 148, p = 0.043), indicating a slight increase in mortality among patients with previous COVID-19 infection. Similarly, ICU stay was weakly but significantly correlated with COVID-19 infection history (Spearman’s ρ = 0.077, p = 0.039), suggesting an increased likelihood of ICU admission in this group. Return to normal work after surgery showed no significant association with prior COVID-19 infection (Spearman’s ρ = 0.058, p = 0.477). For 30-day readmission, a negative correlation was observed (Spearman’s ρ = -0.158), with the result approaching significance (p = 0.054), implying a possible trend toward fewer readmissions in those with a history of COVID-19, though this did not reach statistical significance. Thus, in this hospital-based cohort, previous COVID-19 infection was significantly associated with both postoperative mortality and ICU stay, but not with return to normal work or 30-day readmission (Table 3).

**Table 3:** Association of COVID-19 infection history with postoperative outcomes: Spearman correlation analysis df: degrees of freedom

	COVID 19 Infection History		Spearman Rho	Df	p value
	Yes	No			
Mortality					
Yes	3	3	0.086	148	0.043
No	39	81			
Return to Normal Work					
Yes	39	80	0.058	148	0.477
No	3	4			
30 Days Readmission					
Yes	0	6	-0.158	148	0.054
No	42	76			
ICU Stay					
Yes	3	11	0.077	148	0.039
No	39	73			

Treatment approaches and outcomes for liver abscess cases are shown in Table 4. Most participants received percutaneous drainage (65, 43.3%), followed by antibiotics alone (60, 40%). The majority achieved complete recovery (120, 80%), with minimal cases of recurrence (15, 10%) and a low mortality of 5 (3.3%).

**Table 4:** Treatment and outcomes of patients (n=150) with a liver abscess

Treatment/Outcome	n (150)	%
Type of Treatment		
- Antibiotics Only	60	40
- Percutaneous Drainage	65	43.3
- Open Surgical Drainage	25	16.7
Recurrence of Abscess	15	10
Outcome		
- Complete Recovery	120	80
- Complications (e.g., Septicemia)	20	13.3
- Prolonged Hospitalization (>14 days)	20	13.3
- Mortality	5	3.3

## Discussion

This study included 150 participants diagnosed with a liver abscess, with a mean age of 48.3 years. A male predominance was noted (110, 73.3%), and substantial proportions reported alcohol use (70, 46.7%) and tobacco consumption (45, 30%). A history of COVID-19 infection was documented in 90 (60%) cases, among which 65 (43.3%) required hospitalization and 30 (20%) required ICU admission. Diabetes mellitus and liver cirrhosis were prevalent comorbidities, occurring in 60 (40%) and 35 (23.3%) patients, respectively. Nardo et al. (2021) also reported a similar male predominance in COVID-19-associated liver injury, matching our 110 (73.3%), while ICU admissions in their series were slightly lower at 15% compared to our 30 (20%). The high alcohol use rate in our cohort aligns with their observation that alcohol exacerbates liver-related complications [3]. Similarly, the prevalence of diabetes in our study 40% was consistent with Tian et al. (2020), who reported a 37% rate in COVID-19 patients with liver involvement. Both diabetes and cirrhosis are recognized as contributing to hepatic stress and increased risk of liver abscess formation following COVID-19 [13].

In our cohort, 110 (73.3%) of patients presented with a single abscess, most frequently involving the right hepatic lobe (100, 66.7%). Inflammatory marker analysis revealed elevated CRP (48.3 mg/L) and IL-6 (32.1 pg/mL), indicating pronounced systemic inflammation. Sharma et al. (2021) similarly found that single right-lobe abscesses were most common, attributing this to greater blood flow through the right portal circulation [14]. Elevated CRP and IL-6 levels in our cases, particularly among patients with previous ICU admissions, mirror findings by Chai et al. (2020), who linked these markers to severe COVID-19-related liver injury [5]. Weng et al. (2021) documented post-COVID-19 hepatic enzyme elevations without specifically reporting CRP or IL-6 [14]; our findings add to this by highlighting persistent inflammatory activity in patients with post-COVID-19 liver abscesses.

The correlation analysis revealed that prior COVID-19 infection is significantly, albeit weakly, associated with increased postoperative mortality and ICU admission, indicating that COVID-19 may exacerbate vulnerability to poorer outcomes in subsequent surgical or clinical interventions. However, no significant association was found with return to normal work or 30-day readmission, though there was a trend toward lower readmissions in patients with a COVID-19 history that warrants further investigation. This suggests that while COVID-19 history might impact acute severity and mortality risks, its influence on medium-term recovery milestones is less clear. Weng et al. (2021) further noted gastrointestinal symptoms in 33% of post-COVID-19 patients, with a subset exhibiting liver abnormalities. In comparison, our study identified more severe hepatic manifestations, including cirrhosis in 35 (23.3%) and recurrence of abscess in 15 (10%) cases. This higher burden may reflect the greater proportion of patients with pre-existing liver disease in our cohort, increasing vulnerability to post-COVID-19 complications [14].

Treatment consisted primarily of percutaneous drainage (65, 43.3%) and antibiotic therapy (60, 40%), yielding an overall recovery rate of 120 (80%). Recurrence occurred in 15 (10%) cases, and mortality was low (5, 3.3%). Sharma et al. (2021) reported nearly identical outcomes, with a 78% recovery rate, 8% recurrence, and 3% mortality, underscoring the efficacy of drainage combined with antimicrobial therapy [14]. Tian et al. (2020) emphasized that severe COVID-19 with high inflammatory marker levels is associated with prolonged recovery [13], which was reflected in our 5 (3.3%) patients requiring extended hospitalization due to persistently elevated CRP and IL-6. Lagana et al. (2020) also observed that patients with severe hepatic injury and prior ICU admission often experience longer hospital stays, a trend consistent with our finding that severe COVID-19 history was associated with more complex abscess presentations and prolonged recovery [14].

The limitations of the study include its prospective design, which may introduce bias such as the Hawthorne effect. The absence of a non-liver-abscess comparator group limits the ability to infer causality, restricting conclusions to associations within the liver abscess cohort. Residual confounding remains possible due to incomplete measurement or adjustment for key factors like biliary disease, alcohol use, immunosuppression, recent antibiotic use, and vaccination status. Additionally, microbiological characterization and antibiotic resistance data were limited. Finally, as a single-center study, the findings may have limited generalizability due to local variations in pathogen prevalence and patient demographics.

## Conclusions

In conclusion, this hospital-based study highlights that liver abscess patients with a prior history of COVID-19 infection exhibit a significantly higher risk of postoperative mortality and ICU admission, underscoring the continuing impact of COVID-19 on clinical outcomes. The presence of comorbidities such as diabetes and liver cirrhosis further compounds this risk, emphasizing the need for vigilant monitoring and tailored management in this vulnerable population. Despite these challenges, effective treatment approaches like percutaneous drainage and antibiotics resulted in high recovery rates and low mortality. These findings call for heightened clinical awareness and targeted strategies to improve prognosis in liver abscess patients with a history of COVID-19.
